# xCAPT5: protein–protein interaction prediction using deep and wide multi-kernel pooling convolutional neural networks with protein language model

**DOI:** 10.1186/s12859-024-05725-6

**Published:** 2024-03-10

**Authors:** Thanh Hai Dang, Tien Anh Vu

**Affiliations:** 1 Faculty of Information Technology, VNU University of Engineering and Technology, 144 Xuan Thuy, Hanoi, 10000 Vietnam; 2 Faculty of Biology, VNU University of Science, 334 Nguyen Trai, Hanoi, 10000 Vietnam

**Keywords:** Protein–protein interactions, Convolutional neural networks, Protein language models

## Abstract

**Background:**

Predicting protein–protein interactions (PPIs) from sequence data is a key challenge in computational biology. While various computational methods have been proposed, the utilization of sequence embeddings from protein language models, which contain diverse information, including structural, evolutionary, and functional aspects, has not been fully exploited. Additionally, there is a significant need for a comprehensive neural network capable of efficiently extracting these multifaceted representations.

**Results:**

Addressing this gap, we propose xCAPT5, a novel hybrid classifier that uniquely leverages the T5-XL-UniRef50 protein large language model for generating rich amino acid embeddings from protein sequences. The core of xCAPT5 is a multi-kernel deep convolutional siamese neural network, which effectively captures intricate interaction features at both micro and macro levels, integrated with the XGBoost algorithm, enhancing PPIs classification performance. By concatenating max and average pooling features in a depth-wise manner, xCAPT5 effectively learns crucial features with low computational cost.

**Conclusion:**

This study represents one of the initial efforts to extract informative amino acid embeddings from a large protein language model using a deep and wide convolutional network. Experimental results show that xCAPT5 outperforms recent state-of-the-art methods in binary PPI prediction, excelling in cross-validation on several benchmark datasets and demonstrating robust generalization across intra-species, cross-species, inter-species, and stringent similarity contexts.

**Supplementary Information:**

The online version contains supplementary material available at 10.1186/s12859-024-05725-6.

## Introduction

In the complex cellular environment, proteins regularly interact with each other, forming the foundation for numerous vital biological functions. These interactions, known as protein–protein interactions (PPIs), serve as regulatory hubs for a wide range of cellular processes, including gene expression, cell signaling, and metabolic pathways. To identify and analyze PPIs, various experimental methods have been developed, ranging from high-throughput to low-throughput approaches. Nevertheless, these techniques are often hindered by their high cost, time-intensive nature, and limited accuracy. The field of computational biology has witnessed the emergence of various models for predicting PPIs. These computational approaches have the potential to infer a large number of PPIs with a high degree of accuracy. A substantial portion of these models is focused on predicting PPIs solely through protein sequences. Almost all of them fall into three broad categories, namely model using (i) deep learning solely on protein sequence representations; (ii) deep learning on representations of sequences fused with other information, e.g., 3D structure, network topology, etc.; (iii) conventional machine learning.

Early-stage models of the first category often utilized deep convolutional neural networks and multilayer perceptrons with amino acid embeddings. For example, DPPI [1] employed a deep Siamese-like convolutional neural network with random projection and data augmentation. It utilized PSI-BLAST [2] to extract evolutionary information from protein representations as input. DPPI was known as the first deep learning model to achieve state-of-the-art performance in binary PPI prediction. Another approach, PIPR [3], utilized a Siamese architecture and a residual recurrent convolutional neural network (RCNN) to capture local and sequential features. This provided an automatic multi-granular feature selection mechanism, leading to state-of-the-art performance not only in binary prediction but also in multi-class and affinity prediction. D-SCRIPT [4] is a deep-learning-based model that combines a convolutional neural network (CNN) with a pre-trained language model for the extraction of rich feature representations for each protein. FSNN-LGBM [5] is a hybrid classifier that combines a functional-link-based neural network (FSNN) with a LightGBM boosting classifier. DeepTrio [6] uses a masked multiscale CNN architecture with multiple parallel filters to capture multiscale contextual information from protein sequences.

Regarding the second category, some advanced PPI prediction models have been recently introduced. TAGPPI [7] incorporates sequence features, structural information predicted from AlphaFold, and proteins’ 3D structure features extracted with a graph representation learning method on contact maps. HNSPPI [8] adopts a feature fusion strategy, combining network topology and sequence information for comprehensive feature extraction. It employs a simple classifier for prediction, making it lightweight and efficient. Graph-BERT [9] utilizes a language model-based embedding SeqVec to represent protein sequences and a graph convolutional neural network with the training strategy of subgraph batches using a top-k intimacy sampling approach. The Ensemble Residual Convolutional Neural Network (EResCNN) [10] model integrates multiple feature extraction techniques with a Residual Convolutional Neural Network (RCNN) for predicting protein–protein interactions. It employs an ensemble learning framework that combines RCNN with a tree-based machine learning method, significantly enhancing predictive performance. The MARPPI model [11] is a multi-scale residual network with a dual-channel and multi-feature approach designed for predicting Protein–Protein Interactions (PPIs). It leverages Res2vec for association information between residues, utilizing pseudo amino acid compositions, auto-correlation descriptors, and multivariate mutual information for comprehensive feature extraction. Topsy-Turvy [12] is a model based on D-SCRIPT, which combines both sequence-based and global network-based views of protein interactions. The model incorporates patterns from both views during training, resulting in state-of-the-art performance in PPI prediction.

In addition to deep learning-based approaches, there are still some conventional machine learning-based models (of the third category) recently proposed. These models have demonstrated promising results in predicting binary PPIs. StackPPI [13] combines a rich set of biologically relevant feature encodings with a powerful stacked ensemble classifier consisting of random forest, extremely randomized trees, and logistic regression algorithms. It achieves high predictive accuracy through advanced feature selection and dimensionality reduction using XGBoost. Subsequently, GcForestPPI [14] is a novel deep-forest-based method for predicting PPIs. It leverages an elastic net for optimizing the process of comprehensive feature extraction from pseudo amino acid composition, autocorrelation descriptors, and various position-specific scoring matrices. Its ensemble of XGBoost, random forest, and extremely randomized trees within a cascade architecture significantly outperforms existing predictors.

In this paper, we introduce xCAPT5, a novel model for predicting protein–protein interactions using solely based on protein sequences. xCAPT5 is based on a multi-kernel deep convolutional neural network with a Siamese architecture, followed by XGBoost [15]. Further, xCAPT5 applies the Protein Language Model ProtT5-XL-UniRef50 [16] to capture various aspects of amino acids in a protein sequence, including contextual, physicochemical, evolutionary, and functional information. By using a deep multi-kernel CNN, xCAPT5 captures fine-grained details of individual residues and their neighbors through smaller kernels, as well as broader structural patterns of the protein sequences through larger kernels. A Siamese architecture allows xCAPT5 to capture the interdependency between a pair of protein sequences. During the training phase, xCAPT5 refines these representations, resulting in discriminative latent representations for protein pair interactions. These learned representations are then utilized by XGBoost, an advanced machine learning algorithm that employs an ensemble of decision trees to generate interaction probabilities.

Our contributions are four-fold. Firstly, we introduce xCAPT5, a versatile architecture that is applicable to a broad range of pair-wise prediction problems, including but not limited to the PPI prediction. Secondly, we demonstrate that the incorporation of embeddings based on a protein language model can significantly improve the model performance over traditional embeddings. Thirdly, xCAPT5 establishes a new benchmark in PPI prediction performance across various datasets, outperforming over ten state-of-the-art existing related models in various tasks, including cross-validation and generalized inference across different species and on independent datasets with unseen data. Finally, xCAPT5’s relatively high recall rate in identifying PPIs makes it a powerful tool for investigating interactomes across both well-studied and lesser-known species, underscoring its utility in generalization tasks and offering a valuable tool for the scientific community engaged in the study of protein–protein interactions.

## Methods

### Model architecture

**Fig. 1:**
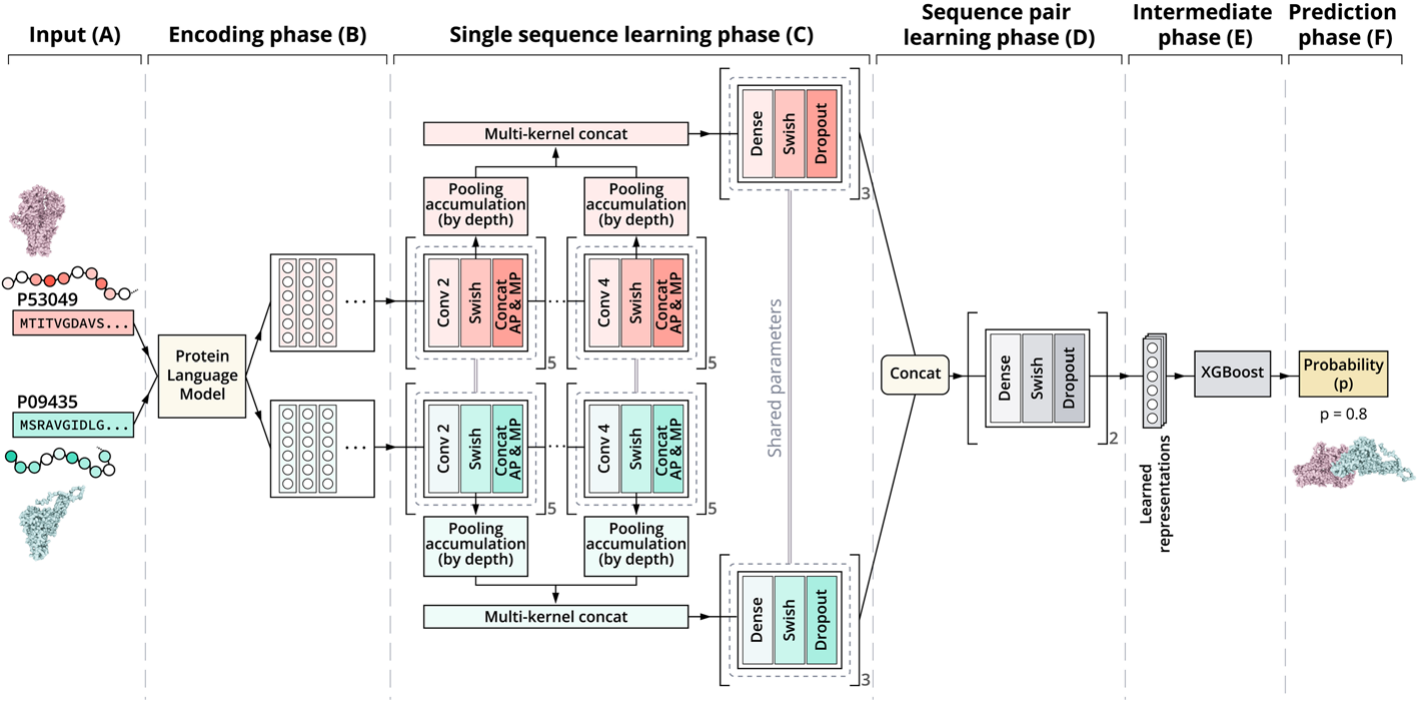
Overview of the xCAPT5 Model Architecture, which encompasses five distinct phases denoted by the capital letters in the parenthesis.** A** Input Stage: The model takes two protein sequences as input.** B** Embedding Phase: The ProtT5-XL-UniRef50 Protein Language Model processes the sequences to produce amino acid embeddings.** C** Single Sequence Learning Phase: Subsequent to embedding, each sequence traverses through five convolutional modules. Within each module, four layers are executed in sequence: the first performs convolutions with kernel sizes 2 (conv 2), 3 (conv 3, not illustrated in the figure), and 4 (conv 4), generating varying feature maps. These maps are then activated via the Swish function in the second layer. The third layer acts on the activation output, applying average pooling (AP) and max pooling (MP) to retain the most important features. The fourth layer (Pooling accumulation by depth), functioning as an auxiliary pathway, applies global max pooling and global average pooling on activation output across different depths, followed by a multi-kernel concatenation (Multi-kernel concat) to create a comprehensive feature profile for each sequence. The concatenated outputs are processed through a two-layer feed-forward network incorporating fully connected layers (dense), ReLU activation, and drop out.** D** Sequence Pair Learning Phase: The extracted representations from individual sequences are combined and fed into a three-layer feed-forward network to learn the refined features of protein pairs.** E** Intermediate Phase: The XGBoost algorithm is employed to train on these integrated features, optimizing the model’s predictive capability.** F** Prediction Phase: The final output is a probabilistic score given by the trained XGBoost model, which predicts the interaction potential between the two input protein sequences

In this section, we present the general architecture of our xCAPT5 model, which consists of two multi-kernel deep convolutional neural networks (CNN) combined within the Siamese architecture and the extremely boosted model XGBoost for the sequence-based binary PPIs prediction. The xCAPT5’s architecture is depicted in Fig. 1. Our model generally encompasses five distinct phases: Amino Acids (AA) encoding, protein sequence learning, protein pair learning, intermediate phase, and prediction. Each phase plays a crucial role in the overall architecture and functionality of xCAPT5.

- The encoding phase encodes protein sequences as amino acid embeddings via the ProtT5-XL-UniRef50 Protein Language Model, adeptly encapsulating a broad spectrum of protein characteristics, including evolutionary trends, physicochemical properties, and structural nuances.
- The sequence learning phase employs two deep convolutional neural networks within the Siamese architecture, utilizing varying kernel sizes to meticulously learn and simultaneously capture each protein sequence’s local and global features.
- The third phase focuses on understanding the mutual influence between protein pairs by concatenating the individual sequence representations and feeding through a deep multi-layer perceptron (MLP). It aims to construct a comprehensive representation of each protein pair’s interactive dynamics.
- The fourth phase serves as an intermediate step, creating a post-training learned representation to be fed into an auxiliary classifier for augmenting the original neural network’s predictive accuracy.
- The prediction phase leverages XGBoost, trained on the refined representation to enhance the model performance through the extreme boosting technique.

By harnessing the power of a protein language model, our xCAPT5 can achieve a nuanced understanding of the complex variances inherent in biological sequences, leading to the generation of more accurate and informative protein representations. Our adoption of multi-kernel convolutional neural networks emphasizes that an increase in the number of kernels substantially enhances the model performance. Further, our research underscores the value of deeper network architectures, which are shown to be more effective in identifying intricate protein sequence patterns, thereby elevating the accuracy of PPI predictions. The integration of both Global Average Pooling and Global Max Pooling within our xCAPT5 model strategically maximizes feature retention, merging the benefits of these pooling methods for enhancing the performance.

#### Single protein sequence learning

Following the encoding phase, the protein sequence learning phase in xCAPT5 delves into extracting and comprehending the intricate patterns and representations inherent within pairs of amino acid embeddings, *X* and $X'$. To achieve this, xCAPT5 employs a Siamese architecture that utilizes deep multi-kernel convolutional neural networks (CNNs) combined with the concatenation of global average pooling (GAP) and global max pooling (GMP).

The Siamese architecture is employed to process two protein sequences simultaneously, capturing their respective patterns and representations in a shared network. This architecture facilitates the learning of the latent relationships and interactions between the individual protein sequences. Within the Siamese architecture, the deep multi-kernel CNNs serve as the backbone for extracting meaningful features from the protein sequences. These CNNs employ multiple convolutional kernels, each with a different size $k \in [2, 3, 4]$, to capture both local and global features. The multi-kernel approach enables the network to explore and learn diverse spatial relationships and motifs within the protein sequences, enhancing its ability to comprehend the complex characteristics embedded within them. To extract and capture the intricate information embedded within the rich-information amino acid embeddings, xCAPT5 constructs deep CNNs corresponding to each kernel size. The deep CNN within xCAPT5 is structured with five blocks consisting of four sequential layers; the number of blocks represent the level of depth (5) in the network.

*The first layer (Convolutional Layer)* applies a set of filters with kernel size *k* to the input *X* (the amino acid embeddings) in the first block or the output of the third layer from the previous block dth $Z_{k}^{d-1}$ with $d\in [1, 5]$, we denote $Z_{k}^{0}:=X$. These filters capture different local patterns and interactions, allowing the network to detect important features within the protein sequences

1 $$\begin{aligned} C_{k}^{d} = \text {Conv}_{k}(Z_{k}^{d-1}), d \in [1,5] \end{aligned}$$

*The second layer (Swish Activation Layer)*, introduces non-linearity into the network via the swish activation function [17]. This function enables the model to capture intricate relationships and dependencies among the learned features effectively. This layer maps the feature maps $C_{k}^{d}$ generated by the convolutional operations in the preceding layer to a set of activated feature maps $Y_{k}^{d}$.

2 $$\begin{aligned} Y_{k}^{d} = \text {swish}(C_{k}^{d})) \end{aligned}$$

*The third layer (concatenation of average pooling (AP) and max pooling (MP))* receives the activated feature maps $Y_{k}^{d}$ as input and performs both AP and MP operations followed by a spatial dropout operation, referred to as SpatialDrop, a regularization technique that randomly deactivates entire feature maps during training to prevent the model from relying excessively on specific spatial locations or local patterns, thereby reducing overfitting. This layer effectively combines global context information derived from AP and the most discriminative local features derived from MP. Following the pooling operations, another spatial dropout operation is applied to further enhance the robustness of the model. The output of this layer is a set of pooled and regularized feature maps $Z_{k}^{d}$.

3 $$\begin{aligned} Z_{k}^{d} = \text {SpatialDrop}\left( \left[ \text {MP}\left( Y_{k}^{d}\right) , \text {AP}\left( Y_{k}^{d}\right) \right] \right) \end{aligned}$$

*The Fourth Layer* *(Pooling Accumulation)*, not a direct layer in the flow of information through the deep CNN, instead it functions as a sidechain module. The GMP (Global Max Pooling) and GAP (Global Average Pooling) operations are applied to the output from the second layer $Y_k^{d}$, producing two vectors that represent the most significant (GMP) and average (GAP) features. These two vectors are then concatenated to form a comprehensive feature map that carries both global and local information about the input, which is then subjected to a dropout operation (denoted by Drop) to reduce overfitting.

4 $$\begin{aligned} G_{k}^{d} = \text {Drop}\left( \left[ \text {GMP}\left( Y_{k}^{d}\right) , \text {GAP}\left( Y_{k}^{d}\right) \right] \right) \end{aligned}$$

Consequently, the vectors $G_{k}^{d}$ that are generated at each depth level are accumulated in a depth-wise manner. This depth-wise accumulation ensures a comprehensive aggregation of information from all levels of the network. As a result, the module efficiently manages and integrates the critical feature information that has been extracted and processed by the previous layers in the deep CNN. This procedure facilitates a depth-wise understanding of the hierarchical representations of the protein sequences, thereby enhancing the model’s ability to interpret and learn from complex protein sequence data.

5 $$\begin{aligned} G_k = \left[ G_k^{1},...,G_k^{5} \right] \end{aligned}$$

After the depth-wise pooling accumulation for each kernel size *k*, the resulting vectors $G_{k}$ are concatenated. This comprehensive representation, denoted as *G*, captures a wide array of features from the input sequences. The vector $G\in \mathbb {R}^{1200}$ is a fusion of information extracted by convolutional layers with different kernel sizes. We apply the batch normalization (BatchNorm) and the dropout operation as follows to make the training more stable and generalize better.

6 $$\begin{aligned} G = \text {Drop}\left( \text {BatchNorm}\left( \left[ G_2, G_3, G_4 \right] \right) \right) \end{aligned}$$

Deep CNN with different multiple kernel sizes working together allows the model to capture different scales of spatial relationships in the input data. Smaller kernel sizes can capture fine-grained, local features, while larger kernel sizes can pick up on more global, abstract features. By concatenating the accumulated vectors for each kernel size, the model can retain and leverage these diverse scales of features simultaneously. Upon capturing the features from the protein sequences through CNNs, these features embodied in the vector *G* are directed into a feed-forward block for further refinement and transformation. This process entails the application of linear transformations along with non-linear activation functions within the feed-forward block. As a result, the model is capable of encapsulating the vital characteristics of the protein sequence more effectively, contributing to a reduction in data dimensionality.

The Siamese architecture ensures that both sequences in the pair go through the same processing steps with shared weights. This means that for the second sequence in the pair, a feature tensor $G'$ is created in the same way as *G* for the first sequence. Both sequences are independently fed through the same deep multi-kernel CNNs, and the extracted features from each are then passed through the same feed-forward sub-network. For each sequence, the output from the feed-forward sub-network is a vector $S\in \mathbb {R}^{186}$ or $S'\in \mathbb {R}^{186}$, depending on whether it’s the first or second sequence in the pair. The feed-forward block comprises three consecutive layers, each with a fully connected layer followed by a swish activation function and dropout. Here, $W_{1} \in \mathbb {R}^{744\times 1200}, b_{1}\in \mathbb {R}^{744}, W_{2}\in \mathbb {R}^{372\times 744}, b_{2}\in \mathbb {R}^{372}, W_{3}\in \mathbb {R}^{186\times 372}, b_3\in \mathbb {R}^{186}$ denote the weights and biases of the first, second, and third layer, respectively.

7 $$\begin{aligned} S_{1}&= \text {Drop}(\text {swish}(W_{1}G+b1)) \end{aligned}$$

8 $$\begin{aligned} S_{2}&= \text {Drop}(\text {swish}(W_{2}S_{1}+b2))\end{aligned}$$

9 $$\begin{aligned} S&= \text {Drop}(\text {swish}(W_{3}S_{2}+b3)) \end{aligned}$$

#### Sequence pair learning

In the sequence pair learning phase, the goal is to capture the dependencies and characteristics that define the interaction between two protein sequences. To achieve this, the processed features of the two sequences, denoted as *S* and $S'$, are combined and fed into a multi-layer perceptron (MLP). This phase is crucial for learning the latent relationships and interactions between the pair, enabling accurate prediction of their interaction. To form a composite feature map, the refined feature vectors *S* and $S'$ are concatenated, resulting in a combined feature map $P = \left[ S_2, S_2'\right] \in \mathbb {R}^{372}$. This composite feature map captures the information from both sequences and their potential mutual information. This concatenated feature map is then passed through a MLP, which is composed of two densely connected layers, each followed by a swish activation function and a dropout operation. Here, $M_{1}\in \mathbb {R}^{328\times 372}, c_{1} \in \mathbb {R}^{328}, M_{2} \in \mathbb {R}^{164\times 328}, c_{2} \in \mathbb {R}^{164}, M_{3} \in \mathbb {R}^{1\times 164}, c_3\in \mathbb {R}$ denote the weights and biases of the first-, the second fully connected layer, and the output layer respectively.

10 $$\begin{aligned} P_{1}&= \text {Drop}(\text {swish}(M_{1}P+c_{1})) \end{aligned}$$

11 $$\begin{aligned} P_{2}&= \text {Drop}(\text {swish}(M_{2}P_{1}+c_{2})) \end{aligned}$$

12 $$\begin{aligned} P_{3}&= M_{3}P_{2}+c_{3}\end{aligned}$$

13 $$\begin{aligned} \text {p}&= \frac{1}{1+e^{-P_{3}}} \end{aligned}$$

These equations illustrate the transformations that the combined feature map undergoes as it is passed through the MLP. The final output of the MLP, represented as $\text {p}$, is obtained by applying a sigmoid function to the output of the final dense layer. This sigmoid function maps the final output to a range between 0 and 1, thus making it interpretable as the probability of interaction between the protein sequence pair.

#### The intermediate phase

Subsequent to the initial training phase of the neural network xCAPT5, the derived representations from xCAPT5 are put into use. Once training is complete, the dataset is passed through xCAPT5 and the model’s penultimate layer representations ($P_{3}$ representation from section “Sequence pair learning”), denoted as *P*, are extracted. These derived representations, *P*, are then fed into an XGBoost [15], a powerful gradient boosting framework, which proceeds to further refine these representations, enhancing the model’s ability to capture complex patterns in the data. This additional layer of processing serves to enhance the model’s overall predictive power and accuracy.

#### Prediction

Once the XGBoost model is fully trained, it can be used to predict PPIs. The model outputs a score for each protein pair, which can be interpreted as the predicted probability of interaction for that pair. A decision threshold is set, often at 0.5, for binary classification tasks. If the predicted probability is greater than this threshold, the model predicts that the pair of sequences interact. If the predicted probability is lower than the threshold, the model predicts that they do not interact. By leveraging the strengths of both deep learning through xCAPT5 and gradient boosting through XGBoost, the model is able to effectively learn from the protein sequence data and accurately predict protein–protein interactions.

Let $P = [p_1, p_2,..., p_n]$ be the learned representations obtained by passing the training dataset through the trained neural network xCAPT5, where *n* is the total number of instances in the dataset. Let $y = [y_1, y_2,..., y_n]$ denote the corresponding labels for these instances. For a given dataset $D = \{ (p_i, y_i) \}$, $(|D| = n, p_i \in \mathbb {R}^m, y_i \in \mathbb {R})$ with *n* instances and *m* features, the prediction process is described as follows:

$$\begin{aligned} {\hat{y}}_i = \sum _{k=1}^{K} f_k(p_i) \end{aligned}$$

where *K* represents the total number of trees and $f_k(p_i)$ represents the prediction score of the learned representation $p_i$ on the $k^{th}$ tree. XGBoost is an ensemble learning method which uses the space of regression trees as its base classifiers, so the prediction score of the XGBoost algorithm can also be expressed by the above formula, and the objective function can be defined as follows:

$$\begin{aligned} Obj(\theta ) = \sum _{i=1}^{n} l(y_i, {\hat{y}}_i) + \sum _{k=1}^{K} \Omega (f_k) \end{aligned}$$

where $l(y_i, {\hat{y}}_i)$ represents the training error of the learned representation $p_i$. In these boosting methods, the $k^{th}$ tree is added to complete the $t^{th}$ iteration and the prediction function is defined as:

$$\begin{aligned} {\hat{y}}_i^{(t)} = \sum _{k=1}^{t} f_k(p_i) = {\hat{y}}_i^{(t-1)} + f_t(p_i) \end{aligned}$$

where ${\hat{y}}_i^{(t)}$ represents the prediction result of the combined *t* tree models on the learned representation $p_i$, the $l(y_i, {\hat{y}}_i^{(t)})$ of the $t^{th}$ tree is a constant, and $\Omega (f_k)$ is used to describe the complexity of the $k^{th}$ tree as the regularizing term, expressed as follows:

$$\begin{aligned} \Omega (f_k) = \gamma T + \frac{1}{2} \lambda \sum _{j=1}^{T} w_j^2 \end{aligned}$$

where $\gamma$ and $\lambda$ are the regularization parameters, and $w_j$ is the score of the leaf nodes. Then the model can be written as $f_t(p) = w^{\textsf{T}} q(p), w \in \mathbb {R}^T$ for each regression tree. *q*(p) indicates the leaf nodes corresponding to the learned representation *p*, and *T* is the number of leaf nodes of the tree.

The first derivative $g_i$ and the second derivative $h_i$ are simultaneously used to approximate the function using Taylor’s expansion. Then the objective function can be converted into the form of the leaf node of the $t^{th}$ tree by combining the above formulas and using the equality $f_t(p) = w^{\textsf{T}} q(p), w \in \mathbb {R}^T$. The solution process is described as follows:

$$\begin{aligned} Obj'(\theta )&= \sum _{i=1}^{n} l(y_i, {\hat{y}}_i^{(t-1)} + f_t(p_i)) + \Omega (f_t) + C \\&\approx \sum _{j=1}^{T} [G_j w_j + \frac{1}{2} (H_j + \lambda ) w_j^2] + \gamma T \end{aligned}$$

where,

$$\begin{aligned} G_j= & {} \sum _{i \in I_j} g_i = \sum _{i \in I_j} \partial _{{\hat{y}}_i^{(t-1)}} l(y_i, {\hat{y}}_i^{(t-1)}) \\ H_j= & {} \sum _{i \in I_j} h_i = \sum _{i \in I_j} \partial ^2_{{\hat{y}}_i^{(t-1)}} l(y_i, {\hat{y}}_i^{(t-1)}) \end{aligned}$$

Then, the optimal weights *w* can be reflected in the first step *g* and the second step *h*, and obtained as follows:

$$\begin{aligned} w_j^* = -\frac{G_j}{H_j + \lambda } \end{aligned}$$

### Model hyperparameters

We use three kernel sizes of 2, 3, 4. For each kernel size, each CNN is designed with a depth of 5 (blocks). The network employs a spatial dropout rate of 0.15 and a standard dropout rate of 0.05 to prevent overfitting and enhance generalization. We configure the hidden layers with 744, 372, and 186 units, while the final multilayer perceptron (MLP) after the merge has 328 and 164 units. For the optimization, we employ the Adam optimizer [18] with learning rate 1e-3, Amsgrad setting [19], epsilon 1e-6, and batch size 64.

Regarding the XGBoost, the gbtree booster is used for utilizing gradient boosting trees. Regularization is applied via a $\text {reg}\_\text {lambda}$ (L2 regularization term on weights) of 1 and an alpha value (L1 regularization term on weights) of 1e-7 to prevent overfitting. Subsampling of the dataset and column sampling by tree are set at 0.8 and 0.2 respectively. The model utilizes 1000 estimators with a maximum tree depth of 5 to ensure a balance between the model complexity and performance. The model also sets a minimum child weight of 2 to avoid overfitting. Furthermore, gamma of 1e-7 is used as a minimum loss reduction parameter and eta of 1e-6 as a learning rate to maintain a slow and steady model learning process.

### Datasets and experiments

In this paper, we did three intensively thorough experiments to evaluate the performance of our proposed model, comparing it with recent state-of-the-art PPI prediction models on several benchmark datasets. The evaluation metrics used were accuracy, precision, recall, specificity, F1-score, and Matthews correlation coefficient (MCC), Area Under the Receiver Operating Characteristic curve (AUROC), and Area Under the Precision-Recall curve (AUPRC).

The first experiment involves evaluating the learning capacity of models by conducting five-fold cross-validation on three golden standard datasets. These datasets include the Martin *H. pylori* dataset [20] with 1458 positive pairs and 1365 negative pairs, the Guo yeast dataset [21] with 5594 positive pairs and 5594 negative pairs, and the Pan human dataset [22] with 27593 positive pairs and 34298 negative pairs.

The second experiment focuses on evaluating the generalized inference capacity of models on three tasks: intra-species inference, cross-species inference, and inter-species inference. For the training phase, we employs two distinct datasets to ensure a comprehensive learning scope: the human Pan dataset, which is characterized by its balanced composition, and the human Sledzieski dataset [4, notable for its unbalanced nature. This strategic dataset choice is designed to test and enhance the models' generalization abilities across varied data distributions. For intra-species evaluation, we use three human PPI datasets from Li’s work [23]: HPRD with 3516 PPIs, DIP with 1468 PPIs, and HIPPIE HQ (high-quality) with 15489 PPIs, and HIPPIE LQ (low-quality) with 101684 PPIs. Cross-species evaluation involves testing the models on datasets from other species, including mouse, fly, yeast, *C. elegans*, and *E. coli*, retrieved from Sledzieski’s datasets [4]. These datasets consist of 5000 positive pairs and 50000 negative pairs, except for the *E. coli* dataset, which has 2000 positive pairs and 20000 negative pairs. The inter-species evaluation focuses on human-other species PPI test datasets from Yang’s work [24]. These datasets are for 8 viruses: HIV (with 9880 positive and 98800 negative pairs), Herpes (5966 and 59660), Papilloma (5099 and 50990), Influenza (3044 and 30440), Hepatitis (1300 and 13000), Dengue (927 and 9270), Zika (709 and 7090), and Sars-CoV-2 (586 and 5860 pairs).

The third experiment involves evaluating the learning capacity of xCAPT5 on more constrained datasets with different stringent similarities in sequences. Chen’s multispecies dataset [3] is used, with stringent similarity values ranging from 0.01 to 0.4. The performance of the models is evaluated using five-fold cross-validation, with higher stringent similarity values indicating more challenging datasets.

Our proposed xCAPT5 model is compared with eleven recent state-of-the-art models, including PIPR (2019) [3], FSNN-LGBM (2021) [5], GCForestPPI (2021) [14], D-SCRIPT (2021) [4], Topsy-Turvy (2022) [12], DeepTrio (2022) [6], TAGPPI (2022) [7], Graph-BERT (2023) [9], HNSPPI (2023) [8], EresCNN (2023) [10] and MARPPI (2023) [11].

## Results

### Cross-validation performance

**Table 1:** 5-Fold cross-validation performances of methods on Martin dataset

Method	Accuracy (%)	Precision (%)	Recall (%)	Specificity (%)	F1-Score (%)	MCC (%)
PIPR (2019)	80.84 ± 0.44	81.44 ± 0.69	81.55 ± 0.85	80.32 ± 0.67	81.43 ± 0.45	61.69 ± 0.89
FSNN-LGBM (2021)	96.49 ± 0.13	96.03 ± 0.26	97.23 ± 0.04	95.69 ± 0.29	96.62 ± 0.12	92.98 ± 0.25
GcForestPPI (2021)	89.26	88.95	89.71	NA	88.33	78.57
MARPPI (2023)	91.80 ± 1.16	90.69 ± 2.68	94.51 ± 1.13	91.22 ± 1.25	NA	83.74 ± 2.32
HNSPPI (2023)	93.21 ± 0.35	88.47 ± 0.53	**99.39 ± 0.21**	NA	93.59 ± 0.32	93.21 ± 0.35
EresCNN (2023)	87.89	87.84	87.96	NA	87.90	75.81
Our xCAPT5	**97.27 ± 0.12**	**97.30 ± 0.24**	97.07 ± 0.20	**97.44 ± 0.11**	**97.18 ± 0.25**	**94.82 ± 0.20**

NA denotes that data is not available. Report with mean and standard deviation. The bold is the best performance in each metric

**Table 2:** 5-Fold cross-validation performances of methods on Guo dataset

Method	Accuracy (%)	Precision (%)	Recall (%)	Specificity (%)	F1-Score (%)	MCC (%)
PIPR (2019)	96.47 ± 0.21	96.31 ± 0.23	96.67 ± 0.22	96.65 ± 0.22	96.48 ± 0.20	92.45 ± 0.42
FSNN-LGBM (2021)	98.46 ± 0.20	98.73 ± 0.25	98.18 ± 0.18	98.74 ± 0.25	98.45 ± 0.20	96.92 ± 0.39
MARPPI (2023)	96.03 ± 0.76	98.12 ± 0.98	93.51 ± 1.22	98.82 ± 0.25	NA	91.83 ± 1.32
TAGPPI (2022)	97.81	98.10	98.26	98.10	97.80	95.63
HNSPPI (2023)	98.57 ± 0.11	98.30 ± 0.22	98.85 ± 0.13	NA	98.57 ± 0.11	NA
Our xCAPT5	**99.76** ± **0.05**	**99.76** ± **0.04**	**99.75** ± **0.07**	**99.77** ± **0.04**	**99.37** ± **0.27**	**99.52** ± **0.10**

NA denotes that data is not available. Report with mean and standard deviation. The bold is the best performance in each metric

**Table 3:** 5-Fold cross-validation performances of methods on Pan dataset

Method	Accuracy (%)	Precision (%)	Recall (%)	Specificity (%)	F1-Score (%)	MCC (%)
PIPR (2019)	98.26 ± 0.02	98.68 ± 0.04	97.40 ± 0.04	97.93 ± 0.03	98.04 ± 0.02	96.49 ± 0.03
FSNN-LGBM (2021)	99.50 ± 0.28	98.48 ± 0.12	99.39 ± 0.54	99.58 ± 0.10	99.43 ± 0.32	98.98 ± 0.57
Graph-BERT (2023)	99.02 ± 0.13	98.94 ± 0.88	99.15 ± 0.95	98.57 ± 1.19	99.04 ± 0.10	98.00 ± 0.28
Our xCAPT5	**99.77** ± **0.02**	**99.75** ± **0.03**	**99.75** ± **0.02**	**99.80 ± 0.02**	**99.62** ± **0.06**	**99.55** ± **0.03**

NA denotes that data is not available. Report with mean and standard deviation. The bold is the best performance in each metric

On the Martin data set (Table 1), xCAPT5 exhibits a consistently superior performance across various performance metrics. The model leads with an outstanding accuracy of 97.27%, significantly 1% higher than its closest competitor, FSNN-LGBM of 96.49%. xCAPT5 also excels in other metrics such as precision of 97.30%, specificity of 97.44%, F1-Score of 97.19%, and Matthews Correlation Coefficient (MCC) of 94.82%. Interestingly, while HNSPPI shows a marginally better recall score of 99.39%, it falls short in other metrics like precision and MCC. This suggests that while HNSPPI is excellent at identifying true positives, it may not be as well-rounded as xCAPT5, which exhibits high performance in multiple metrics simultaneously.

Experimental results on the Guo data set demonstrate that xCAPT5 outperforms all compared models by significant margins across multiple key metrics. With a remarkable accuracy of 99.76%, xCAPT5 eclipses its nearest competitor, HNSPPI, which scored 98.57% in accuracy (Table 2). In terms of precision, xCAPT5 maintains its dominion with a score of 99.76%, compared to FSNN-LGBM’s 98.73%, once again indicating superior specificity. The model’s recall rate is 99.75%, making it the leader in identifying true positive cases as well; the closest competitor here is HNSPPI at 98.85%. The same trend is evident in the specificity, F1-score, and Matthews Correlation Coefficient (MCC) categories, where xCAPT5 posts scores of 99.77%, 99.37%, and 99.52%, respectively.

Furthermore, on the Pan dataset (Table 3), xCAPT5 significantly outperforms its closest competitors across all metrics, showcasing an accuracy of 99.77% with an exceptionally low standard deviation of 0.02%. The closest competitor, FSNN-LGBM, has a slightly lower accuracy of 99.50% but with a notably higher standard deviation of 0.28%, indicating less consistent results. The gap between xCAPT5 and its competitors is also significant. While FSNN-LGBM lags by a narrow margin of 0.27% in accuracy, this difference is amplified by the variation indicated by standard deviations. In precision, recall, and other metrics, xCAPT5 consistently ranks highest, almost always surpassing the 99.5% threshold with minimal variance.

**Table 4:** Statistical significance of accuracy differences between xCAPT5 and other models across three datasets

Model	Martin Dataset	Guo Dataset	Pan Dataset
MARPP (2023)	$6.32 \times 10^{-5}$	$9.20 \times 10^{-4}$	NA
TAGPPI (2022)	NA	$8.20 \times 10^{-8}$	NA
HNSPPI (2023)	$1.81 \times 10^{-8}$	$1.55 \times 10^{-5}$	NA
PIPR (2019)	$1.81 \times 10^{-8}$	$8.90 \times 10^{-7}$	$1.23 \times 10^{-5}$
Graph-BERT (2023)	NA	NA	$1.23 \times 10^{-5}$
FSNN-LGBM (2021)	$1.13 \times 10^{-3}$	$1.79 \times 10^{-8}$	$7.76 \times 10^{-2}$

NA denotes that data is not available for the comparison

Statistical analyses of the models’ performance across three distinct datasets reveal that xCAPT5 consistently outperforms other methods in terms of accuracy. Specifically, the adjusted *p-*values, derived from Welch’s t-tests [25] and controlled for false discovery rate using the Benjamini-Hochberg procedure [26], underscore the statistical significance of these results (Table 4). In the Martin dataset, both MARPP and FSNN-LGBM show p-values indicating significant differences, yet the extremely low p-values for HNSPPI and PIPR suggest an even more pronounced difference in accuracy compared to xCAPT5. For the Guo dataset, TAGPPI and FSNN-LGBM exhibit highly significant improvements with p-values reaching $8.20 \times 10^{-8}$ and $1.79 \times 10^{-8}$ respectively. Similarly, PIPR also shows a significant difference in this dataset. Notably, the comparisons on the Pan dataset are limited but still present compelling evidence of xCAPT5’s superior accuracy, with Graph-BERT showing a significant difference, although FSNN-LGBM does not exhibit a statistically significant variation.

### Generalized inference evaluation

We evaluated the generalization capacity of xCAPT5 and compared models by training them on human-centric data sets and subsequently testing them on independent datasets. Our assessment encompasses a diverse range of test scenarios, spanning intra-species (human), cross-species (model organisms), and inter-species (human-virus) PPI datasets. The foundational training on human datasets equipped the models with discern patterns and features intrinsic to human protein interactions. By subjecting them to disparate test datasets, we aimed to ascertain the models’ proficiency in extrapolating their predictions beyond the confines of their training data. This rigorous analysis offers insights into the models’ competence in reliably predicting PPIs across varied biological contexts. Furthermore, it paves the way for the potential extrapolation of these models to species with scant or non-existent PPI data. In scenarios where specific PPI data is absent but protein sequence information is available, the models’ foundational training on human datasets can be harnessed to facilitate informed predictions.

#### Intra-species inference

The intra-species inference analysis presents the evaluation results of different methods on two distinct training datasets: the balanced training dataset Pan and the imbalanced training dataset Sledzieski. The performance of the methods is measured in terms of recall percentage on various test datasets.

Additional file 1: Table S1 shows the evaluation results for the intra-species dataset trained on the balanced Pan dataset. Across all test datasets (HPRD, DIP, HIPPIE HQ, HIPPIE LQ), xCAPT5 consistently achieves the highest recall. For instance, on the HPRD dataset, xCAPT5 achieves a recall of 96.16%, outperforming both PIPR (91.95%) and FSNN-LGBM (94.28%). The same trend is observed for other test datasets as well, with xCAPT5 consistently outperforming the other methods. xCAPT5 ranks first in terms of recall percentage for all of these datasets.

Additional file 1: Table S2 presents the evaluation results for the intra-species dataset trained on the imbalanced Sledzieski dataset. Despite the imbalance in both the training dataset and the test datasets, xCAPT5 again demonstrates superior performance. It achieves the highest recall on most test datasets. For example, on the DIP dataset, xCAPT5 achieves a recall of 67.64%, surpassing the recall of PIPR (30.79%) and FSNN-LGBM (48.71%). It achieves the highest recall on most test datasets, including HPRD, DIP, and HIPPIE HQ. However, it is worth noting that Topsy-Turvy achieves a slightly higher recall of 51.22% on the HIPPIE LQ dataset compared to xCAPT5’s 40.92%.

#### Cross-species inference

The cross-species inference analysis shows the evaluation performance of different methods on cross-species datasets trained on two different training sets: Pan and Sledzieski. The test datasets represent various species:* E. coli*, Fly, Mouse, Worm, and Yeast.

In Additional file 1: Table S4, where models are trained on the balanced training set Pan, we observe varying performance across the different methods and test datasets. D-SCRIPT consistently demonstrates the highest Precision, with values ranging from 70.64% (Yeast) to 85.47% (Mouse). It also achieves competitive F1-Scores, ranging from 33.88% (Yeast) to 53.68% (Fly), indicating a good balance between Precision and Recall. D-SCRIPT also performs well in terms of AUROC and AUPRC, achieving high values in most test datasets. Our model xCAPT5 shows the highest Recall values in several test datasets, such as Fly (83.08%) and Worm (71.02%). However, its Precision is relatively lower compared to D-SCRIPT.

In Additional file 1: Table S3, where models are trained on the unbalanced training set Sledzieski, we can observe a decrease in overall performance compared to the first table. The Precision values of all methods are generally lower, indicating a higher number of false positives. However, xCAPT5 still shows the highest Precision, ranging from 9.18% (*E. coli*) to 9.45% (Yeast). Notably, the Recall values are consistently high across all methods and test datasets, ranging from 85.62% (Yeast) to 99.55% (*E. coli*) for xCAPT5.

#### Inter-species inference

In Additional file 1: Table S5, the evaluation inference performance of our proposed model xCAPT5 and compared models on inter-species datasets trained on the balanced training set Pan is presented. The test datasets include Dengue, HIV, Hepatitis, Herpes, Influenza, Papilloma, SARS-CoV-2, and Zika.

Experimental results indicate that xCAPT5 generally performs the best across different test datasets. For example, in the Dengue test dataset, xCAPT5 achieves a precision of 9.21%, recall of 97.19%, F1-score of 16.83%, AUROC of 50.73%, and AUPRC of 9.44%. Our model demonstrates competitive performance across most test datasets. It achieves the highest Precision on the Hepatitis and Papilloma datasets and the highest Recall on the HIV dataset. Additionally, xCAPT5 achieves the highest F1-Score on the Zika dataset.

In Additional file 1: Table S6, the evaluation inference performance of different methods on inter-species datasets trained on the unbalanced training set Sledzieski is presented. The test datasets are the same as in the S5. Experimental results indicate that xCAPT5 performs well in most test datasets. For example, in the Dengue test dataset, xCAPT5 achieves a precision of 23.36%, recall of 35.66%, F1-score of 28.22%, AUROC of 54.90%, and AUPRC of 14.71%. Among the compared models, xCAPT5 consistently outperforms others in terms of Precision, Recall, and F1-Score on most test datasets. Notably, xCAPT5 achieves the highest Precision on the Hepatitis and Herpes datasets and the highest Recall on the HIV and Hepatitis datasets. It also obtains the highest F1-Score on the HIV and Influenza datasets.

### Stringent similarity evaluation

In this section, we assess the ability of our proposed model xCAPT5 to generalize to datasets with varying constraints on sequence similarity (Table 5). xCAPT5 stands out with its exceptional performance. It consistently achieves an accuracy of 99.72% and an F1 score of 99.61% across various sequence identities. This performance remains stable even when the sequence identity threshold tightens from 40% to just 1%. Such consistency indicates that xCAPT5 consistently delivers accuracy rates above 99.70% and F1 scores over 99.50%.

On the other hand, while PIPR, TAGPPI, and DeepTrio show commendable results, there’s a noticeable pattern: their performance metrics slightly decrease as the sequence identity requirements become stricter. This indicates that these models might face challenges when adapting to less familiar sequence spaces. The fluctuations in accuracy and F1-Score of xCAPT5 are minimal, with the most significant change being a mere 0.06% in accuracy. This consistent performance, even under tightening sequence similarity constraints, underscores xCAPT5’s robustness and superior generalization capabilities. Unlike many models that might falter under strict conditions, xCAPT5’s resilience is evident, suggesting that it’s adept at handling a broad spectrum of sequence identities without significant performance degradation.

**Table 5:** 5-Fold cross-validation performances of methods on stringent Chen multispecies datasets

Similarity	Methods	Accuracy (%)	F1-Score (%)
Any	PIPR (2019)	98.19	98.17
	DeepTrio (2022)	98.20	98.20
	TAGPPI (2022)	99.15	99.15
	Our xCAPT5	**99.72**	**99.61**
$\le 40\%$	PIPR (2019)	98.29	98.28
	DeepTrio (2022)	97.83	97.98
	TAGPPI (2022)	99.10	99.16
	Our xCAPT5	**99.76**	**99.60**
$\le 25\%$	PIPR (2019)	97.91	98.08
	DeepTrio (2022)	97.52	97.75
	TAGPPI (2022)	98.99	99.06
	Our xCAPT5	**99.74**	**99.61**
$\le 10\%$	PIPR (2019)	97.54	97.79
	DeepTrio (2022)	97.32	97.62
	TAGPPI (2022)	98.97	99.08
	Our xCAPT5	**99.70**	**99.53**
$\le 1\%$	PIPR (2019)	97.51	97.80
	DeepTrio (2022)	97.11	97.47
	TAGPPI (2022)	98.89	98.89
	xCAPT5	**99.73**	**99.60**

Report with mean. The bold is the best performance in each metric

### Hyperparameter effect

In this section, we assess the impact of hyperparameters on the performance of the xCAPT5 model, with a specific focus on the neural network architecture of xCAPT5. We employed a 5-fold cross-validation method on the Guo dataset to assess the neural architecture of xCAPT5 under different hyperparameter configurations.

We note that increasing the number of kernel sizes from 2 to 3 leads to a significant performance improvement across multiple metrics. This suggests that a wider range of kernel sizes enables the model to detect a broader spectrum of patterns in the input data, enhancing overall performance. However, further increasing to four results in a decline in performance (Additional file 1: Figure S1). This deterioration can be attributed to increased complexity, making it harder for xCAPT5 to learn and generalize effectively. The model becomes more susceptible to capturing noise and irrelevant details, hindering its ability to discern relevant patterns and leading to decreased performance.

The depth of a Convolutional Neural Network (CNN), traditionally defined by the number of layers, plays a pivotal role in the model’s learning capacity. However, in the context of the xCAPT5 model, the depth is uniquely characterized by the number of blocks, with each block representing a level of depth. The xCAPT5 model is composed of five such blocks, signifying a depth of five. As the depth of the network increases, denoted by the number of blocks in the xCAPT5 model (Additional file 1: Figure S2), there is a corresponding improvement in the model’s performance. The optimal performance is observed when the network comprises five blocks. This optimal depth is influenced by certain parameters, such as the padding of the sequence length to 1200 and the use of a pooling size of 4.

Furthermore, our investigation encompasses the comparison of xCAPT5’s performance using different amino acid embeddings. In this regard, we discovered that leveraging the large protein language models like ProtT5-XL-U50, ProtT5-XL-BFD, ProtBert-BFD [16], and PlusRNN [27] provides superior results compared to traditional approaches like one-hot encoding and physicochemical concatenated with Skip-Gram embedding (Additional file 1: Figure S3). This highlights the importance of incorporating advanced protein language models in enhancing the predictive capabilities of xCAPT5. Our examination also reveals that a hybrid model, which combines a machine learning algorithm with a neural network, yields a marked performance enhancement (Additional file 1: Figure S4). Specifically, the integration of a machine learning model leads to a significant accuracy increase of nearly 10% for the Martin dataset, approximately 2% for Guo, and just under 1% for Pan, highlighting the substantial benefits of this approach over a standalone neural network model.

## Discussion

In this study, we examine the design of models for predicting protein–protein interactions (PPIs) solely based on protein sequences. This approach is grounded in the hypothesis that protein sequences inherently contain sufficient information for PPI prediction, a concept increasingly recognized in contemporary research. Our methodology is notable for being among the pioneering efforts to apply a deep and wide convolutional neural network to amino acid embeddings derived from a protein language model for PPI prediction. This approach has demonstrated notable efficacy, outperforming traditional methods that rely on embeddings from non-protein language models, including those based on universal amino acid embeddings such as Skip-Gram or one-hot encoding, as well as protein sequence embeddings utilizing generic protein feature descriptors. The use of a protein language model facilitates a nuanced comprehension of the intricate variations and complex characteristics of biological sequences. This results in more precise and informative protein representations. Notably, among various protein language models evaluated, ProtT5-XL-UniRef50 emerged as the most effective, showcasing superior predictive performance in our analyses.

Our use of multi-kernel CNNs marks a significant departure from previous studies that relied on single-kernel networks. We found that increasing the number of kernels enhances the model performance, suggesting that a multi-kernel design is beneficial in this context. Additionally, our study also reveals that deeper network architectures correlate with the improved performance, effectively capturing complex protein sequence patterns and boosting protein–protein interaction predictions. Furthermore, the integration of Global Average Pooling and Global Max Pooling in our xCAPT5 model optimizes the retention of crucial features, combining the strengths of both pooling methods. On top of that, we leveraged the strengths of a neural network for representation extraction, followed by feeding these learned features into a machine learning algorithm. This strategy effectively boosted the overall performance of our model, capitalizing on the neural network’s ability to extract nuanced features and the machine learning algorithm’s proficiency in utilizing these features for enhanced outcomes.

In our comprehensive evaluations, the xCAPT5 model underwent cross-validation against three gold-standard benchmark datasets, confirming its robustness and reliability—a cornerstone practice for machine learning model validation. xCAPT5 demonstrated state-of-the-art performance, with accuracy rates of 97.27%, 97.76%, and 99.77% on the Martin, Guo, and Pan datasets, respectively. Considering the field’s maturity and the multitude of advanced models already in existence, the notable achievement of xCAPT5 surpassing the second-best models by an average of 1% across all metrics marks a significant stride in predictive model development. The average standard deviation of xCAPT5 across the datasets is the lowest at 0.06%, indicating the most stable performance among all models considered.

To rigorously evaluate the xCAPT5 model’s ability to learn and infer across different biological contexts, we adopted balanced Pan and unbalanced Sledzieski training set on humans and then tested on unseen data categorized into intra-species (human PPIs across four datasets), cross-species (PPIs from five other model organism datasets), and inter-species (human and virus PPIs from eight datasets). xCAPT5 consistently outperformed the compared SOTA models on most test datasets regardless of the training dataset, showing exceptional generalization capabilities in predicting PPIs across these categories. This is particularly noteworthy in the context of computational biology research, where models often face diverse datasets and training sets.

The experimental analysis of the xCAPT5 model, focusing on generalized inference capabilities, reveals notable distinctions in performance based on the nature of the training dataset. When trained on the balanced Pan dataset, xCAPT5 exhibits a remarkably high recall rate, averaging around 95.50%. This is particularly significant in biological research, where the comprehensive detection of protein–protein interactions is crucial, especially in contexts where only genomic data are available, such as in lesser-studied species. The high recall indicates the model’s proficiency in identifying true positive interactions, a critical aspect in exploring the interactome of these species. In contrast, training on the unbalanced Sledzieski dataset results in lower recall but higher precision, F1-Score, AUROC, and AUPRC. This suggests a more refined accuracy in the predictions, albeit with a possible trade-off in missing certain interactions. The choice between these training approaches depends on the research objectives: high recall is vital for exploratory studies aiming to map unknown interactomes comprehensively, while higher precision and balanced metrics are preferable for validating specific hypotheses or in well-characterized research areas.

In the context of protein–protein interaction predictions, the stringent similarity evaluation of xCAPT5 is particularly important as it addresses a key challenge: the decline in model performance with decreasing sequence similarity. Typically, as sequence similarity lowers, the prediction task becomes more challenging, adversely affecting most models’ accuracy. However, xCAPT5 demonstrates a notably smaller decline in performance compared to other models like PIPR, TAGPPI, and DeepTrio, maintaining high accuracy (99.73%) and F1-scores (99.60%) even at the challenging $\le 1\%$ similarity threshold.

While the xCAPT5 model excels in generalization and robustness across various datasets, it’s important to recognize its limitations. Particularly, in handling unbalanced datasets, such as Sledzieski, it is observed that models like Topsy-Turvy and D-SCRIPT surpass xCAPT5 in the Precision metric, highlighting a critical area where the xCAPT5’s Recall-oriented approach may compromise its efficacy, especially in scenarios where minimizing false positives is paramount. This inclination towards recall over precision reflects a strategic trade-off that might not align with the demands of applications requiring stringent accuracy.

Additionally, the model’s performance fluctuates with the class imbalance, signaling a potential gap in its capacity to uniformly manage diverse dataset characteristics. Moreover, the complexity of xCAPT5 could hinder interpretability, a crucial aspect in fields necessitating transparency and understanding of predictive mechanisms. The model’s intricate architecture necessitates extensive hyperparameter tuning, which can be a complex and time-intensive process, potentially hindering rapid development and deployment. Finally, the model emphasizes prediction accuracy without providing insight into the discriminative quality of its internal representations, such as the absence of analysis on how these representations cluster when subjected to dimensionality reduction techniques like PCA. This omission suggests a gap in the exploration of the underlying feature space that the model has learned.

## Conclusion

Our research introduces xCAPT5, a groundbreaking classifier that harnesses the power of the T5-XL-UniRef50 protein language model to produce rich amino acid embeddings from protein sequences. At its heart, xCAPT5 utilizes a multi-kernel deep convolutional siamese neural network, adept at capturing complex interaction features on both micro and macro scales. This is further enhanced by integrating the XGBoost algorithm, which significantly boosts the classification performance of protein–protein interactions (PPIs). xCAPT5 stands out by concatenating max and average pooling features depth-wise, allowing it to learn vital features while maintaining low computational costs. This study marks one of the first attempts to leverage informative amino acid embeddings from a large protein language model through a deep and wide convolutional network. The experimental results are compelling, showing that xCAPT5 surpasses recent state-of-the-art methods in binary PPI prediction. Its exceptional performance is consistent across various tests, including cross-validation on multiple benchmark datasets and robust generalization in intra-species, cross-species, inter-species, and stringent similarity contexts.

### Supplementary Information

**Additional file 1**. Supplementary Materials for xCAPT5.

## Data Availability

xCAPT5 is available in the GitHub repository at https://github.com/aidantee/xCAPT5.
