# Supplementary material for: xCAPT5: protein–protein interaction prediction using deep and wide multi-kernel pooling convolutional neural networks with protein language model
Source: BMC Bioinformatics. 2024 Mar 10;25:106. doi: 10.1186/s12859-024-05725-6 (PMC10924985; doi:10.1186/s12859-024-05725-6)
Supplement: Supplementary file 1 — Additional file 1. Supplementary Materials for xCAPT5. [file 12859_2024_5725_MOESM1_ESM.pdf]

Supplementary data for xCAPT5: Protein-Protein Interaction Prediction Using Deep and Wide Multi-kernel Pooling Convolutional Neural Networks with Protein Language Model

Thanh Hai Dang[1*] and Tien Anh Vu[2††]

[1*]Faculty of Information Technology, VNU University of Engineering and Technology, 144 Xuan Thuy, 10000, Hanoi, Vietnam.
[2]Faculty of Biology, VNU University of Science, 334 Nguyen Trai, 10000, Hanoi, Vietnam.

*Corresponding author(s). E-mail(s): hai.dang@vnu.edu.vn;
Contributing authors: vutienanh_t63@hus.edu.vn;
†These authors contributed equally to this work.

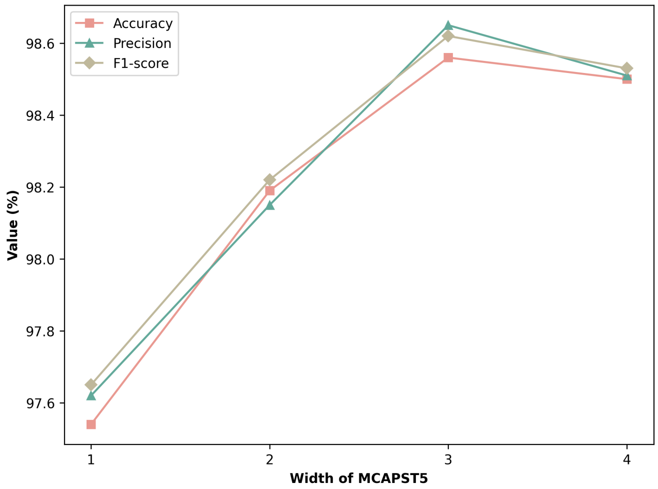

Figure S1. Evaluation of Hyperparameter Width Impact on xCAPT5 Model Performance

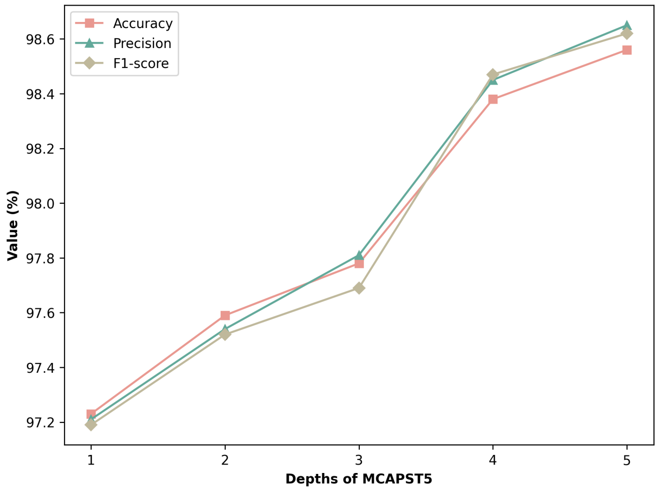

Figure S2. Evaluation of Hyperparameter Depth Impact on xCAPT5 Model Performance

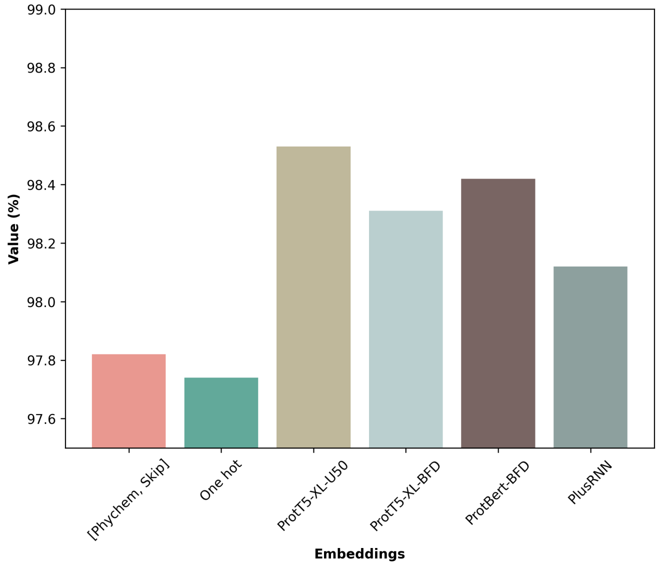

Figure S3. Evaluation of Hyperparameter Embedding Impact on xCAPT5 Model Performance

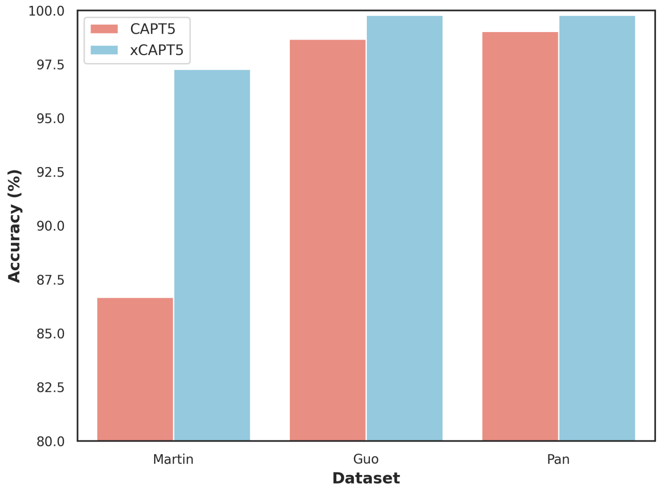

Figure S4. Performance Comparison of Neural Networks (CAPT5) and Hybrid Models (xCAPT5)

Table S1: Intra-species Dataset Inference Performance: Analysis of Methods Trained on the Pan Dataset

**Test datasets**	**Methods**	**Recall (%)**
HPRD	PIPR	91.95
	FSNN-LGBM	94.28
	xCAPT5	**96.16**
DIP	PIPR	93.46
	FSNN-LGBM	93.33
	xCAPT5	**96.75**
HIPPIE HQ	PIPR	91.23
	FSNN-LGBM	91.29
	xCAPT5	**94.55**
HIPPIE LQ	PIPR	92.26
	FSNN-LGBM	91.92
	xCAPT5	**93.92**

Report with mean

Table S2: Intra-species Dataset Inference Performance: Analysis of Methods Trained on the Sledzieski Dataset

**Test datasets**	**Methods**	**Recall (%)**
HPRD	PIPR	22.84
	FSNN-LGBM	40.93
	D-SCRIPT	12.62
	Topsy-Turvy	51.22
	xCAPT5	**58.53**
DIP	PIPR	30.79
	FSNN-LGBM	48.71
	D-SCRIPT	11.44
	Topsy-Turvy	56.67
	xCAPT5	**67.64**
HIPPIE HQ	PIPR	32.24
	FSNN-LGBM	46.41
	D-SCRIPT	12.54
	Topsy-Turvy	45.19
	xCAPT5	**58.84**
HIPPIE LQ	PIPR	22.83
	FSNN-LGBM	38.54
	D-SCRIPT	7.23
	Topsy-Turvy	**51.22**
	xCAPT5	40.92

Report with mean

Table S3: Cross-species Dataset Inference Performance: Analysis of Methods Trained on the Pan Dataset

**Test datasets**	**Methods**	**Precision (%)**	**Recall (%)**	**F1-Score (%)**	**AUROC (%)**	**AUPRC (%)**
	PIPR	8.91	90.21	16.22	46.88	8.53
E. Coli	FSNN-LGBM	8.67	92.14	15.84	47.51	8.73
	xCAPT5	**9.18**	**99.55**	**16.79**	**50.47**	**9.17**
	PIPR	8.59	87.70	15.65	44.39	8.13
Fly	FSNN-LGBM	8.74	92.59	15.98	48.01	8.78
	xCAPT5	**9.29**	**96.70**	**16.95**	**51.13**	**9.71**
	PIPR	8.72	89.76	15.89	46.78	8.53
Mouse	FSNN-LGBM	8.85	93.12	16.16	48.59	8.86
	xCAPT5	**9.36**	**97.46**	**17.07**	**51.52**	**9.35**
	PIPR	8.61	90.24	15.73	43.35	7.98
Worm	FSNN-LGBM	8.81	93.63	16.11	48.36	8.83
	xCAPT5	**9.31**	**96.50**	**16.98**	**51.24**	**9.30**
	PIPR	8.52	85.62	15.50	43.83	8.03
Yeast	FSNN-LGBM	8.86	93.29	16.19	48.68	8.88
	xCAPT5	**9.45**	**94.78**	**17.18**	**51.98**	**9.43**

Report with mean

Table S4: Cross-species Dataset Inference Performance: Analysis of Methods Trained on the Sledzieski Dataset

**Test datasets**	**Methods**	**Precision (%)**[1]	**Recall (%)**	**F1-Score (%)**	**AUROC (%)**[2]	**AUPRC (%)**
E. Coli	PIPR	55.81	17.30	26.41	70.85	31.59
	FSNN-LGBM	19.10	24.95	21.63	57.19	11.59
	D-SCRIPT	**75.54**	38.30	50.83	**86.03**	**53.54**
	Topsy-Turvy	39.81	46.40	42.85	64.30	37.22
	xCAPT5	44.41	**64.95**	**52.75**	78.41	32.03
Fly	PIPR	52.17	14.44	22.62	73.21	29.56
	FSNN-LGBM	22.62	30.42	25.95	60.01	13.21
	D-SCRIPT	**81.56**	40.00	53.68	84.17	59.69
	Topsy-Turvy	63.01	75.62	68.74	90.07	**67.66**
	xCAPT5	62.77	**83.08**	**71.51**	**91.08**	53.69
Mouse	PIPR	67.06	34.94	45.94	85.08	52.53
	FSNN-LGBM	32.05	51.56	39.53	70.32	20.93
	D-SCRIPT	**85.47**	34.48	49.14	79.92	54.91
	Topsy-Turvy	59.03	71.04	64.48	86.41	**60.80**
	xCAPT5	53.14	**84.78**	**65.33**	**87.64**	54.35
Worm	PIPR	62.81	15.64	25.04	76.54	34.80
	FSNN-LGBM	25.49	29.50	27.35	60.44	13.93
	D-SCRIPT	**85.03**	32.98	47.53	80.21	56.31
	Topsy-Turvy	70.55	63.85	67.04	85.20	**62.98**
	xCAPT5	77.52	**71.02**	**74.13**	**86.48**	59.69
Yeast	PIPR	37.71	10.56	16.50	71.30	22.68
	FSNN-LGBM	22.27	24.76	23.45	58.06	12.36
	D-SCRIPT	**70.64**	22.28	33.88	78.89	40.46
	Topsy-Turvy	48.32	54.36	51.16	76.20	43.14
	xCAPT5	62.87	**58.82**	**60.78**	**79.67**	**44.72**

Report with mean

Table S5: Inter-species Dataset Inference Performance: Analysis of Methods Trained on the Pan Dataset

**Test datasets**	**Methods**	**Precision (%)**[1]	**Recall (%)**	**F1-Score (%)**	**AUROC (%)**[2]	**AUPRC (%)**
Dengue	PIPR	7.59	92.88	16.77	**51.35**	9.35
	FSNN-LGBM	9.09	96.18	16.61	50.02	9.09
	xCAPT5	**9.21**	**97.19**	**16.83**	50.73	**9.44**
HIV	PIPR	**9.18**	92.71	16.72	**51.41**	**9.36**
	FSNN-LGBM	8.86	91.49	16.15	48.67	8.88
	xCAPT5	9.21	**94.34**	**16.78**	50.41	9.16
Hepatitis	PIPR	**9.12**	93.03	16.62	**51.84**	**9.49**
	FSNN-LGBM	9.10	**96.71**	**16.63**	50.03	9.09
	xCAPT5	8.78	89.70	15.99	48.24	8.81
Herpes	PIPR	8.93	93.56	16.31	48.51	8.84
	FSNN-LGBM	8.99	95.37	16.44	49.45	9.01
	xCAPT5	**9.21**	**95.83**	**16.81**	**50.68**	**9.32**
Influenza	PIPR	**9.12**	**97.01**	**16.67**	**50.62**	**9.20**
	FSNN-LGBM	8.97	94.13	16.38	49.32	8.98
	xCAPT5	8.97	93.66	16.37	49.29	8.97
Papilloma	PIPR	9.06	94.84	16.54	47.49	8.64
	FSNN-LGBM	9.09	96.74	16.62	50.01	9.09
	xCAPT5	**9.17**	**97.06**	**16.74**	**50.43**	**9.17**
SARS-CoV-2	PIPR	8.99	96.13	16.44	**50.76**	**9.25**
	FSNN-LGBM	**9.14**	**97.57**	**16.71**	49.29	9.14
	xCAPT5	9.03	93.48	16.48	49.98	9.06
Zika	PIPR	9.01	95.35	16.46	48.94	8.89
	FSNN-LGBM	9.23	91.05	16.75	50.72	9.21
	xCAPT5	**9.76**	**95.89**	**17.71**	**50.97**	**9.42**

Report with mean

Table S6: Inter-species Dataset Inference Performance: Analysis of Methods Trained on the Sledzieski Dataset

Test datasets	Methods	Precision (%)[1]	Recall (%)	F1-Score (%)	AUROC (%)[2]	AUPRC (%)
Dengue	PIPR	24.68	6.87	10.62	**62.31**	15.32
	FSNN-LGBM	18.31	33.82	24.02	59.67	12.33
	D-SCRIPT	17.98	1.45	2.68	61.25	13.16
	Topsy-Turvy	22.01	22.71	22.35	61.08	**16.14**
	xCAPT5	**23.36**	**35.66**	**28.22**	54.90	14.71
HIV	PIPR	24.25	9.10	13.19	63.91	16.02
	FSNN-LGBM	17.57	32.58	22.83	58.64	11.86
	D-SCRIPT	19.46	4.63	7.47	50.11	10.96
	Topsy-Turvy	16.87	7.19	10.08	53.44	11.04
	xCAPT5	**27.14**	**36.10**	**30.98**	**64.20**	**17.61**
Hepatitis	PIPR	20.84	1.96	3.58	51.42	11.03
	FSNN-LGBM	15.94	21.46	18.09	54.94	10.49
	D-SCRIPT	**32.68**	0.97	1.89	**59.43**	12.92
	Topsy-Turvy	20.61	7.87	11.39	50.99	10.24
	xCAPT5	20.71	**22.46**	**21.55**	56.93	**13.70**
Herpes	PIPR	22.88	6.21	9.74	56.89	**12.94**
	FSNN-LGBM	16.06	**26.87**	20.10	56.41	10.97
	D-SCRIPT	**27.54**	0.90	1.75	**58.96**	12.74
	Topsy-Turvy	18.51	8.39	11.53	50.36	10.55
	xCAPT5	22.23	21.79	**22.01**	57.08	12.35
Influenza	PIPR	21.62	6.16	9.43	62.54	14.42
	FSNN-LGBM	18.63	**34.65**	24.22	59.75	12.39
	D-SCRIPT	14.06	1.19	2.19	56.17	11.49
	Topsy-Turvy	19.82	13.01	15.66	55.91	11.55
	xCAPT5	**22.67**	30.80	**26.12**	**63.17**	**16.61**
Papilloma	PIPR	16.62	2.92	4.94	55.61	11.37
	FSNN-LGBM	16.43	**26.33**	20.23	56.47	11.02
	D-SCRIPT	**22.87**	1.47	0.92	52.13	10.67
	Topsy-Turvy	15.95	8.58	11.14	51.60	10.35
	xCAPT5	20.12	24.12	**21.93**	**58.32**	**13.04**
SARS-CoV-2	PIPR	13.11	3.58	5.56	55.92	11.13
	FSNN-LGBM	15.47	**27.41**	19.77	56.23	10.84
	D-SCRIPT	**21.48**	1.21	2.33	59.02	13.08
	Topsy-Turvy	15.79	7.52	10.19	55.56	10.59
	xCAPT5	17.90	22.89	**20.09**	**60.29**	**14.81**
Zika	PIPR	13.59	6.96	8.77	57.21	12.57
	FSNN-LGBM	15.56	**30.84**	**20.67**	57.09	11.15
	D-SCRIPT	**42.47**	7.27	12.40	**69.06**	**20.57**
	Topsy-Turvy	14.72	19.40	16.72	57.03	12.17
	xCAPT5	16.59	25.39	20.07	51.90	13.56

Report with mean
